# A Way to Predict Gold Nanoparticles/Polymer Hybrid Microgel Agglomeration Based on Rheological Studies

**DOI:** 10.3390/nano9101499

**Published:** 2019-10-21

**Authors:** Coro Echeverría, Carmen Mijangos

**Affiliations:** Institute of Polymer Science and Technology (ICTP-CSIC), C/Juan de la Cierva 3, 28006 Madrid, Spain

**Keywords:** polymer microgels, hybrid microgels, thermoresponsive, rheology, scaling theory, fractal analysis

## Abstract

In this work, a detailed rheological study of hybrid poly(acrylamide-*co*-acrylic acid) P(AAm*-co-*AAc) aqueous microgel dispersions is performed. Our intention is to understand how the presence of gold nanoparticles, AuNP, embedded within the microgel matrix, affects the viscoelastic properties, the colloidal gel structure formation, and the structure recovery after cessation of the deformation of the aqueous microgel dispersions. Frequency sweep experiments confirmed that hybrid microgel dispersions present a gel-like behavior and that the presence of AuNP content within microgel matrix contributes to the elasticity of the microgel dispersions. Strain sweep test confirmed that hybrid microgels aqueous dispersion also form colloidal gel structures that break upon deformation but that can be recovered when the deformation decreases. The fractal analysis performed to hybrid microgels, by applying Shih et al. and Wu and Morbidelli’s scaling theories, evidenced that AuNP significantly affects the colloidal gel structure configuration ending up with the formation of agglomerates or microgel clusters with closer structures in comparison to the reference P(AAm*-co-*AAc) aqueous microgel dispersions.

## 1. Introduction

Among all colloidal systems, the sub micrometer-sized hydrogel (microgels) particles are of special interest [1,2]. After decades of research, polymeric microgels have revealed their versatility from both the functionality (responsiveness) and applications perspective. These smart materials have received much attention owing to their environmentally tunable sizes and potential applications, such as chemical separation, catalysis, sensors, enzyme immobilization, drug delivery systems, biomimicking artificial synovial fluids, tissue mimicking, and injectable 3D cell scaffolds, among others [3,4,5,6,7,8]. Besides the mentioned advanced applications, microgels are used as building blocks to create structures such as colloidal crystals, films, and gels in the macroscopic scale [9,10,11,12], and more recently as active sites confined within electrospun polymer fibers toward the design of tailored multifunctional stimuli-responsive advanced materials [13,14,15,16]. In our previous works, we first developed poly(acrylamide-*co-*acrylic acid) microgels featured with the ability to swell upon heating, thus showing a positive thermosensitivity and an upper critical solution temperature-like (UCST) volume phase transition temperature [17,18]. This thermo-responsiveness derived from the presence of acrylic acid that forms hydrogen bonds with acrylamide. The obtained UCST-like microgels were characterized in terms size, shape, and thermoresponsiveness, so that the effect of the composition–crosslinking degree and acrylic acid comonomer content could be understood. Moreover, rheological behavior of aqueous poly (acrylamide-acrylic acid) microgel dispersions was also studied from the perspective of a colloidal system. This study outlined their macroscopic elasticity showing that the material behaves as a colloidal gel [17,18].

As deduced from the literature, the evolution of microgel systems advanced toward the development of hybrid systems (organic/inorganic systems). In fact, since the first work of Antonietti et al. [19], in which microgels were used as microreactor and “exo-templates” for the controlled growth of gold nanoparticles, the development of hybrid microgels has increased significantly [20,21,22,23,24,25,26,27,28]. This interest is related to the fact that the incorporation of inorganic nanoparticles into polymeric microgels provided additional functionalities to the final system [29,30]. For instance, the incorporation of gold nanoparticles to thermoresponsive microgels would provide optical properties and thus dual-stimuli responsiveness; the system could be remotely activated (swollen) via light [23].

The intention with this work, besides understanding the effect of adding a nanoparticle into a nano/microgel in the flow behavior, is to propose our approach as a tool to control/predict the formation, breakage, and reformation of agglomerates in colloidal dispersion that might influence their final applications, in particular as drug delivery systems, carriers, or similar. For such purpose, in the present work a detailed rheological study of hybrid P(AAm*-co-*AAc) microgel dispersions is presented. Thus, the aim of this research is to understand how the presence of gold nanoparticles AuNP, that are embedded within the P(AAm*-co-*AAc) microgel matrix, could modify the viscoelastic properties of the aqueous microgel dispersions and their macroscopic elasticity. In previous studies, we could determine that P(AAm*-co-*AAc) aqueous microgels dispersion present a gel-like behavior associated not only to the gel nature of the polymeric microgels themselves, but also to the formation of certain structure due to interactions occurring between microgel particles within the dispersion. Therefore, in this work we will determine if the presence of AuNP within microgels could modify such interactions and hence the described gel-like behavior. To do so, we used two scaling models (Shih et al. [31] and Wu and Morbidelli [32]) to perform a fractal analysis of the hybrid aqueous microgel dispersions.

## 2. Materials and Methods

### 2.1. Materials

As monomer and comonomer we used acrylamide (AA, 99% pure, Sigma-Aldrich, St. Quentin Fallavier, France) and acrylic acid (AAc, Sigma-Aldrich), respectively. To obtain crosslinked microgels N,N’-methylenebisacrylamide (MBA, Sigma-Aldrich, 99.5% pure) was used as crosslinking agent. The reaction was initiated using 2,2’-azobis(2-methylpropionamidine)dihydrochloride (AMPA-d, Sigma-Aldrich, 97% pure). As part of the organic phase, span 80 (sorbitan monooleate) (Fluka, Saint Louis, MO, USA) and dodecane (Fluka, 99% pure) were used as surfactant and organic solvent, respectively. Gold nanoparticles (AuNP) used for their encapsulation into microgel matrix were purchased from Nanogap, which are already covered with poly(n-vinyl-2-pyrrolidone) so that they can be stable in water dispersion. According to Nanogap, AuNP contain 16% gold and the size is approximately 5 ± 1 nm (See Figure S2). All the water used in the preparation and characterization of microgels was Millipore Milli Q grade.

### 2.2. Synthesis of Hybrid P(AAm-AAc)-AuNP Microgels

Microgel synthesis was performed by means of inverse emulsion polymerization (w/o) method as described in [27]. The aqueous phase of the emulsion is formed by acrylamide monomer, acrylic acid comonomer, crosslinker, water dispersed gold nanoparticles, and distilled water (See Table 1 for details). For the oil phase, emulsifier (SPAN 80) and the organic solvent (dodecane) were mixed. Prior to the reaction, both solutions were purged with nitrogen during 30 min. Then, we incorporated the organic phase into a three-necked round bottom flask. Over this organic phase we added the aqueous phase solution by means of a peristaltic pump with a feeding rate of 1.5 mL/min while the forming emulsion was mechanically stirred at 475 r.p.m. This procedure derived in the formation of aqueous phase droplets dispersed in the organic phase. These droplets act as reservoir where the radical polymerization reaction occurs. Finally, the polymerization was thermally initiated using a 2,2’Azo-bis-(2-methylpropionamidine-dihydrochloride (AMPA-d) solution. (The reaction took place at 50 °C, which is the decomposition temperature of the initiator.) Immediately after adding the initiator, the emulsion became turbid. From this stage, the polymerization reaction was allowed to continue for 3 h under nitrogen atmosphere. After this period of time, the reaction was cooled down to room temperature while the stirring and nitrogen flow was maintained to avoid aggregation. Finally, all the prepared emulsions were purified by removal of organic phase by decantation and the remaining aqueous phase was further precipitated in ethanol with subsequent washing by centrifugation at 4500 r.p.m. All samples were redispersed in deionized water and placed in dialysis bags (molecular weight cut off = 3500) for 1 week to remove any unreacted materials. For the sake of clarity, in Table 1 the recipe and reaction conditions for the hybrid microgel synthesis are described.

### 2.3. Characterization Methods

The morphological analysis of the microgels was performed by scanning electron microscopy, SEM (ESEM, XL30, Philips, North Billerica, MA, USA) to determine the shape, size, and dispersion of microgels. Transmission Electron Microscopy, TEM (JEM 3000F, 300kv, JEOL, Tokyo, Japan) was used to confirm the presence and distribution of AuNP within microgel matrix. For this analysis, dried microgels were further dispersed in acetone (We used acetone in order to promote a rapid evaporation of the solvent.). A drop of the dispersion was deposited in a glass wafer, waited till the solvent evaporates, and sputter-coated with gold to minimize charging at fixed conditions for SEM analysis. For TEM analysis a drop of the dispersion was deposited in formvar/carbon-coated grids.

For the rheological study of the hybrid microgel dispersions ARG2 (TA Instrument, New Castle, DE, USA) stress-controlled rheometer was used. First, we determined the linear viscoelastic range by performing strain sweep tests at a constant and non-destructive frequency of 0.5 Hz for hybrid microgel aqueous dispersion with concentrations of 1, 2, and 5 wt%. Then, we carried out frequency sweep tests at a constant strain of 1%, within the linear viscoelastic region. All measurements were performed at 20 °C, the temperature at which microgels are in their collapsed state [27] and using a 60 mm acrylic parallel plate. The temperature sweep tests were performed, from 1 to 40 °C, at a non-destructive frequency of 0.5 Hz, and at a constant strain of 3%. The protocol used for loading the sample were the same for all the aliquots. We used a 1 mL micropipette to add the same volume. The plastic tip used for the loading was previously cut in order to avoid any unwanted deformation prior to the measurement. Microgel dispersions were squeezed the same, by selecting the same gap for all the microgel dispersions and controlling the normal force exerted to each dispersion, so that the initial state is the same for all the samples. In order to preserve or not affect structural equilibrium of the microgels dispersions, we did not perform any preshear prior to the strain sweep test. To ensure a reproducibility and to obtain an average of the determined parameters we performed a minimum of 5 measurements of each microgel dispersion. To determine the critical strain from the stain sweep tests, we selected the second point that comes out of the linearity that coincides with the >5% deviation rule. Fractal analysis was carried out by the application of two scaling theories: Shih et al. [31] and Wu and Morbidelli [32].

## 3. Results and Discussion

### 3.1. Morphology

**Figure 1:**
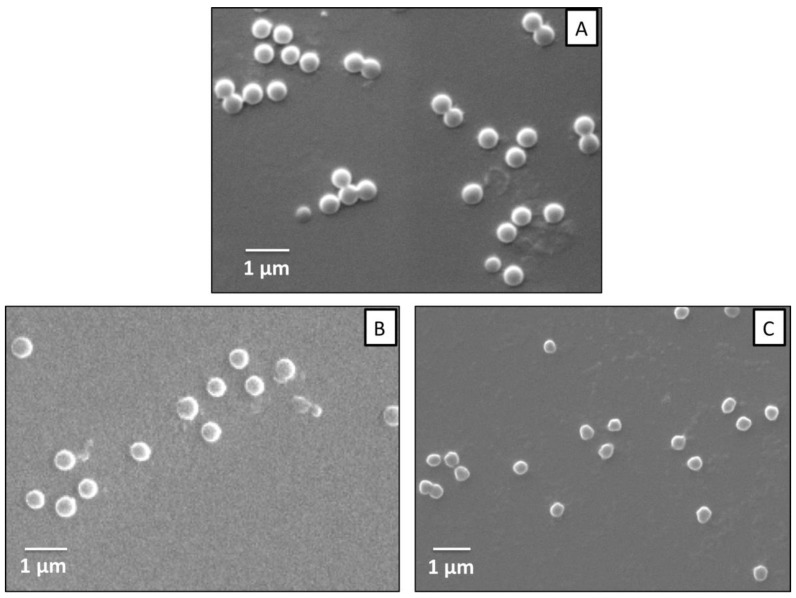
Representative SEM micrographs corresponding to the samples (**A**) P(AAm-*co*-AAc), (**B**) P(AAm-*co*-AAc)-5% AuNP, and (**C**) P(AAm-*co*-AAc)-10% AuNP.

Figure 1 shows SEM (A, B, C) micrographs corresponding to P(AAm-*co*-AAc) microgels and hybrid microgels containing 5% and 10% of AuNP. As observed, the synthesized P(AAm-*co*-AAc) microgels are of spherical shape with diameters in the range of 200 to 500 nm (Figure 1A), which is in agreement with the definition of microgel: intramolecularly crosslinked polymer particle with diameter size in the range of 100 nm to 1 µm. The incorporation of AuNP did not affect the morphology of P(AAm-*co*-AAc) microgels as seen in both Figure 1B,C and already envisaged in previous work [27]. The hybrid microgels are also of spherical shape with similar diameter sizes. Therefore, in terms of morphology, microgels shape and size were not affected by the addition of AuNP, probably due to the small quantity of added nanoparticles and their small size (5 nm) as well. Nevertheless, SEM technique does not allow to detect AuNP.

**Figure 2:**
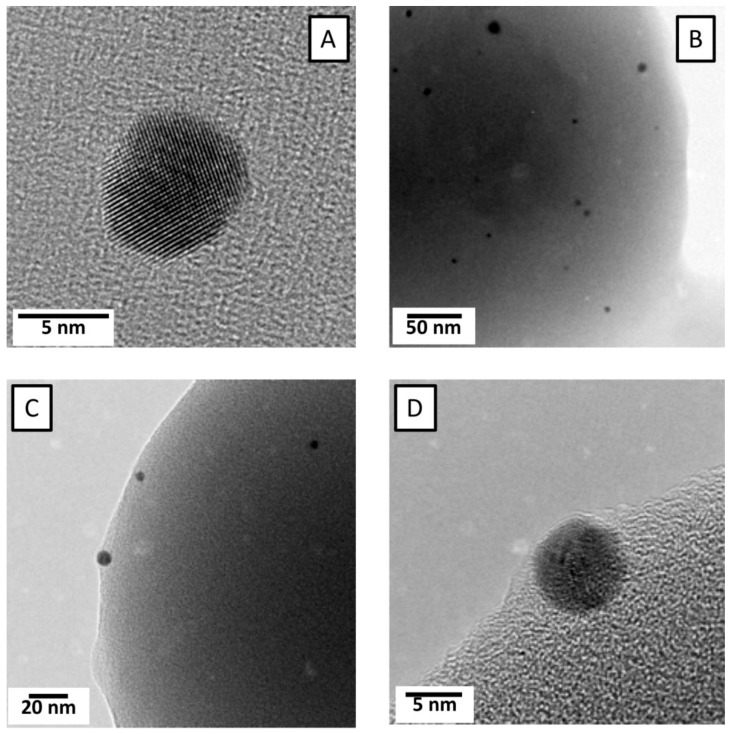
A to D figures show TEM images corresponding to the sample P(AAm*-co-*AAc)-10% AuNP (Adapted from [27] with permission from, Wiley-VCH, Copyright 2010).

In order to confirm the successful encapsulation of AuNP, TEM micrographs corresponding to P (AAm-*co-*AAc)-10% AuNP hybrid microgel sample (Figure 1D,E) are depicted in Figure 2A–D. Those micrographs, reprinted with permission of Elsevier, were taken from our previous work [27]. As shown in Figure 2A, AuNP appear as black spheres in an organic matrix. In particular, in Figure 2A it is possible to observe the atomic planes of the Au nanoparticles, besides confirming that the particles diameter is approximately 5 ± 1 nm. In Figure 2B the image of a single hybrid microgel with black spheres can be observed. Such image is not clear enough to identify whether AuNP are located inside the microgel matrix or on the surface. But if we focus on the Figure 2C,D (magnified from C), near the edge of a single microgel AuNP particles (black spheres) clearly embedded inside the microgel matrix can be observed. This result confirmed the successful encapsulation of AuNP [27]. In order to determine the final AuNP content we also performed thermogravimetric analysis. From TGA experiments we could conclude that the samples P(AAm-*co*-AAc)-5% AuNP and P(AAm-*co*-AAc)-10% AuNP have a final AuNP content of 3% and 8%, respectively. (See Figure S1 and Table S1 of supporting information).

Regarding the influence of AuNP in the swelling ability and thermo-responsiveness, in a previous work we demonstrated that the incorporation of AuNP shifted the volume phase transition temperature of P(AAm*-co-*AAc) towards temperatures close to 37 °C [27].

As mentioned in the introductory section of the manuscript, the versatility of polymeric microgels make them very useful for a wide and diverse spectrum of applications. In this particular case, we developed this hybrid dual responsive microgel system so that it could be potentially used as drug carriers for a further controlled drug release [18]. Therefore, it is crucial to understand the rheology of the microgel aqueous dispersions so that their application could not be conditioned due to undesirable or uncontrollable agglomerations that could occur when applied by injection, for instance, or when circulating in the blood stream so that blood clot could be induced. Taking this into consideration, in this work we put effort in determining the rheological properties of these hybrid microgels so that we could better understand their structure-properties-applications relationship.

### 3.2. Effect of the Incorporation of AuNP in the Viscoelastic Properties of Aqueous Hybrid Microgel Dispersions

**Figure 3:**
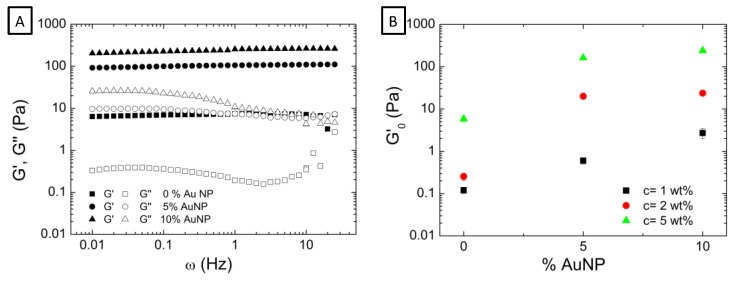
(**A**) Evolution of the elastic (G’) and viscous modulus (G’’) with frequency for P(AAm-*co*-AAc), P(AAm-*co*-AAc)-5% AuNP and P(AAm-*co*-AAc)-10% AuNP microgel aqueous dispersions at a single concentration of 5 wt%. (From 10 to 1 Hz measurement was performed under a constant strain of 3% and from 1 to 0.01 Hz under a constant value of 1% strain). (**B**) Elastic modulus plateau (G’0) represented as a function of AuNP content, for the three different concentration of microgel dispersion: 1, 2, and 5 wt%.

Our first step in the rheological characterization of microgel dispersion was to determine their viscoelastic behavior. Figure 3A shows representative frequency sweep tests corresponding to P(AAm-*co*-AAc), P(AAm-*co*-AAc)-5% AuNP, and P(AAm-*co*-AAc)-10% AuNP aqueous microgel dispersion (C = 5 wt%). As general behavior, the three microgel samples possess the elastic modulus (full symbols) higher than the viscous modulus (empty symbols) at all the frequency ranges studied. Having G’ > G’’ indicates that microgel dispersion presents a solid-like behavior. Moreover, microgel dispersions also present an elastic modulus G’ which is constant and independent of the frequency (showing a finite value at zero frequency). These are the two conditions that define a polymer gel rheologically. Therefore, these two facts together serve to affirm that microgel dispersions present a gel-like behavior as it was also the case of similar systems [17,25]. In addition to these two conditions, the three microgel samples described a minimum in the frequency dependence of G’’ (Figure 3A), which is characteristic of colloidal gels, as stated by Mewis and Wagner [33]. This minimum is better observed in the graph corresponding to the P(AAm-*co*-AAc) microgel (without AuNP).

For a deeper analysis of the results, in Figure 3B we have collected the elastic modulus plateau G’0 (extrapolated at zero frequency) obtained from the frequency sweep tests performed for hybrid microgel dispersions at three different concentrations (1, 2, and 5 wt%), and represented as a function of AuNP content within the microgel matrix. Two results can be drawn: First, that the elastic modulus increases as the concentration of the aqueous microgel dispersions increases, and second and probably more relevant, that the encapsulation of AuNP within the microgel matrix contributes to increase the elastic modulus of the dispersion. For instance, if we analyze the effect of AuNP content in the dispersions containing 5 wt% of microgel, it is observed that the encapsulation of 5% of AuNP within microgel matrix provokes an increase of the G’ modulus one order of magnitude. When the encapsulated AuNP increases from 5% to 10%, the elastic modulus increases also the double, that is, it evolves from approximately 150 Pa (5% AuNP) to 260 Pa (10% AuNP). This result clearly indicates that AuNP act as a filler reinforcing the microgel matrix. The aqueous microgel dispersions’ elastic character is increased giving rise to a stronger gel-like system with improved viscoelastic properties.

**Figure 4:**
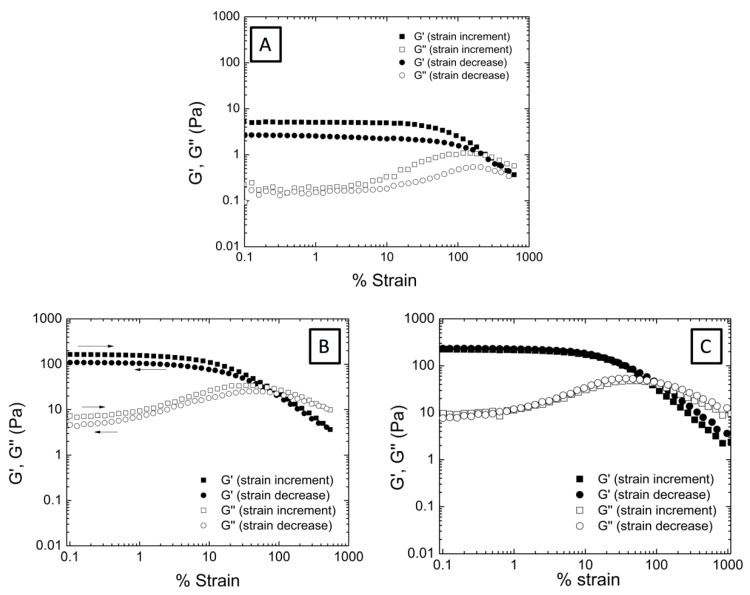
Evolution of G’ (full symbols) and G’’ (empty symbols) as a function of % strain determined as the strain increases (square) and as the applied strain decreases to the initial state (circle) for (**A**) P(AAm-*co*-AAc), (**B**) P(AAm-*co*-AAc)-5% AuNP and (**C**) P(AAm-*co*-AAc)-10% AuNP microgel aqueous dispersions at 5 wt% concentration.

We have confirmed that the encapsulation of AuNP contributes to the elasticity of the system deriving in a stronger gel-like behavior compared to pure microgel dispersions. In previous studies we could determine that the gel-like behavior was associated not only to the gel nature of the polymeric microgels but also to the formation of certain structure (a colloidal gel) due to interactions occurring between microgel particles within the aqueous dispersion [17,25]. In the present work we have studied how hybrid microgel dispersions behave under strain (deformation) and evaluated the influence of AuNP embedded in the microgel matrix. Accordingly, in Figure 4A the evolution of G’ and G’’ with increasing applied strain and for decreasing applied strain is evaluated for P(AAm-*co*-AAc) (5 wt% microgel concentration).

If we analyze the obtained graphs, at low strains both G’ and G’’ keep constant being G’ > G’’. This first stage defines the linear viscoelastic range (LVR). But as the strain increases, both G’ and G’’ reach the limit of linearity at a critical strain value (γ0), becoming dependent on the strain. At certain strain value the modulus crossover occurs (G’ = G’’). From this point on, viscous modulus is higher than the elastic modulus, which is indicative of the liquid-like behavior of the microgel dispersion. When the applied strain is reduced up to the initial value, both G’ and G’’ start to increase again until recovering their independency against the strain ending up in the gel-like behavior described before. The fact that aqueous microgel dispersions are able to recover their initial gel-like behavior implies the formation of some kind of structure that breaks upon deformation that restructure when the deformation disappears [34,35,36]. However, the obtained absolute values of G’ and G’’ are slightly lower in the upturn measurement, showing an incomplete recovery or hysteresis.

When analyzing the strain dependent behavior of the hybrid microgels shown in Figure 4B,C, a similar trend is observed. Hybrid microgel dispersions described a region in the curve at which G’ and G’’ are independent of the applied strain, but as the strain increases both G’ and G’’ become dependent, the cross-over point is achieved and G’, G’’ end up decreasing. When the strain is reduced, hybrid microgel dispersions recover the initial G’, G’’ values, and therefore, the initial gel-like behavior. Interestingly, the encapsulation of 5% of AuNP within microgel matrix derived in weakened hysteresis. But even more remarkable is the behavior described for the P(AAm-*co*-AAc)-10% AuNP microgel dispersions where no hysteresis is observed. This means that, P(AAm-*co*-AAc)-10% AuNP hybrid microgel dispersion is capable to totally recover the initial colloidal gel structure, and therefore the interactions that held the structure (cluster).

In fact, strain dependence behavior (increasing and further decreasing the applied strain) corresponding to P(AAm-*co*-AAc)-10% AuNP shows two superimposed curves indicating that there is no loss of elasticity; G’ and G’’ recover from the imposed deformation. Therefore, hybrid microgel dispersions are capable to totally recover the interactions between microgels and thus the initial colloidal gel structure.

### 3.3. Fractal Analysis of Aqueous Hybrid Microgel Dispersions by Means of Shi et al. and Wu and Morbidelli Scaling Theory: Effect of AuNP in the Microgel Interactions

At this stage, from the evaluation of the viscoelastic properties we have confirmed the reinforcement role that AuNP have in the microgel. Additionally, the analysis of the hybrid microgel dispersions behavior under strain put in evidence the formation of some kind of structure that breaks upon deformation and with the ability to recover as the applied deformation decreases gradually. Previous research regarding the rheological behavior of aqueous dispersions of poly (acrylamide-acrylic acid) microgels already outlined their macroscopic elasticity showing that the material behaves as a colloidal gel [17,18]. These two results are relevant for further potential applications that might imply flow of aqueous dispersions. Indeed, shear-thinning of a colloidal suspension could enable a more homogeneous and easy delivery of the material in the case of injectable materials [37]. And if this behavior is complemented with the total recovery of the elastic properties immediately after injection/deformation, this may prevent the flow of the colloidal solution and facilitate that the material remains on the target site.

There are several studies regarding the interplay of microgel inter-particle interaction modifications by changing particle size, surface charge (functionalization), and crosslinking degree [38,39,40,41,42] (most of them used poly(N-isopropylacrylamide) PNIPAM microgels), but reports aimed to evaluate, control, and predict its influence on the fractal structure and cluster formation through rheology are scarce. For instance, Liao et al. [8,43] generated an in-situ formed hydrogel, using PNIPAM microgels as the building blocks to construct injectable thermal gelling scaffold for 3D cell culture, in the presence of Ca^2+^ to induce changes in the inter-particle interaction. They studied their fractal structure and concluded that both salt concentration and temperature modify the interactions among microgels [8,43]. Recently, we also attempt to modify microgels colloidal behavior through quaternization but no relevant differences were obtained [44]. Therefore, to determine the flow behavior of the hybrid microgel dispersions and to understand how hybrid microgels interact within the aqueous dispersion could be as relevant as the characterization of their responsiveness. Being so, we took advantage of the scaling theories developed by Shih et al. [31] and Wu and Morbidelli [32] and used them as a tool to perform a fractal analysis. These two scaling theories, which are an extension of the computer model proposed by Brown and Ball [45] are based on the fact that microgel dispersions are a collection of flocs–fractal objects closely packed throughout the sample-. These models were recently used to study the interactions occurring among poly(N-Isopropylacrylamide) polycationic microgels [44].

Shih et al.’s [31] models differentiated two extreme situations and unravel the intra- and inter-floc interactions in two separate regimes: strong-link regime where inter-floc interactions are stronger than intra-floc (among particles) interactions and weak-link regime where the elasticity is driven by the mechanically weaker part of the system, that is the weak links between flocs. Shih et al. described each regime based on the scaling relationship of both the elastic constant (elastic modulus plateau) and critical deformation with concentration as shown in the following equations:

(i) Strong-link regime:

(1) $$G_{0}^{\prime }∼\varphi^{\left(d+x\right)/\left(d-D_{f}\right)}$$

(2) $$\gamma_{0}∼\varphi^{-\left(1+x\right)/\left(d-D_{f}\right)}$$

being *φ* the concentration, *d* the Euclidean dimension of the system (*d* = 3), *D_f_* the fractal dimension, and *x* is the fractal dimension of the aggregated backbone that has to be lower than the fractal dimension and positive (*D_f_* > x > 0) [31,46], being a reasonable value 1–1.3 [31].

(ii) Weak-link regime:

(3) $$G_{0}^{\prime }∼\varphi^{\left(d-2\right)/\left(d-D_{f}\right)}$$

(4) $$\gamma_{0}∼\varphi^{1/\left(d-D_{f}\right)}$$

Wu and Morbidelli extended the Shih et al. model to the case where samples belong to a transition regime between the strong and weak link regimes. Therefore, in order to gather these two regimes in a single model, they introduced a new parameter, microscopic elastic constant, *α*. This parameter describes a range of values from 0 to 1, where *α* = 0, corresponds to Shih et al.’s strong-link regime whereas *α* = 1 matches the weak-link regime. Wu and Morbidelli defined the model with the following equations:

(5) $$G_{0}^{\prime }∼\varphi^{\beta /\left(d-D_{f}\right)}$$

(6) $$\gamma_{0}∼\varphi^{\left(d-\beta -1\right)/\left(d-D_{f}\right)}$$

(7) β=(d−2)+(2+x)(1−α)

being *β* an auxiliary parameter that relates α with *x*.

**Figure 5:**
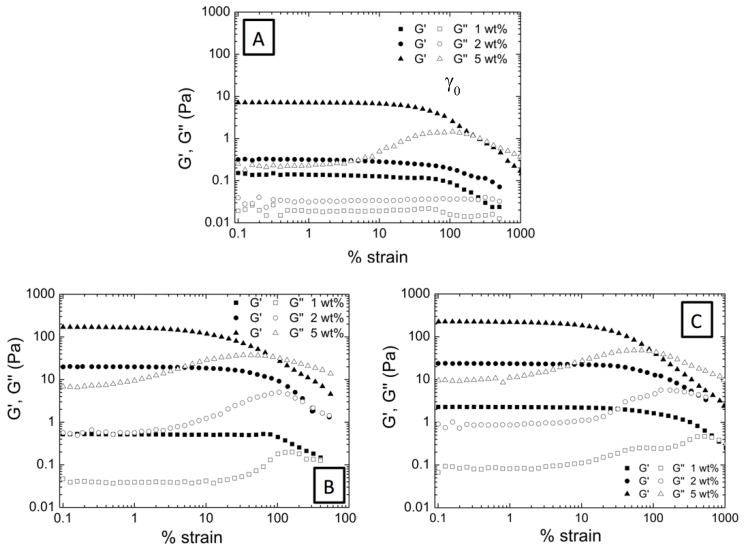
Strain sweep test corresponding to (**A**) P(AAm-*co*-AAc), (**B**) P(AAm-*co*-AAc)-5% AuNP, and (**C**) P(AAm-*co*-AAc)-10% AuNP microgel aqueous dispersions at three different concentrations: 1, 2, and 5 wt%.

In order to perform the fractal analysis, strain sweep tests of hybrid microgels aqueous dispersions were carried out at three different concentrations (1, 2, and 5 wt%) as shown in Figure 5: P(AAm*-co-*AAc) (Figure 5A), P(AAm*-co-*AAc)-5% AuNP (Figure 5B), and P(AAm*-co-*AAc)-10% AuNP (Figure 5C). As observed from the figure, the three samples describe an increase of G’ and G’’ with the microgel concentration. However, the increase of microgel concentration lead to a decrease of the linear viscoelastic range; less strain is necessary to break the formed structure within the dispersion.

**Figure 6:**
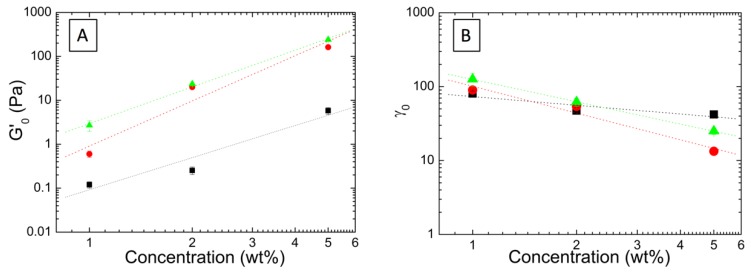
Double-logarithmic plot of (**A**) the elastic modulus plateau, G’_0_, and (**B**) critical strain, γ_0_, as a function of the aqueous microgel concentration for the samples: P(AAm-*co*-AAc) (black square), P(AAm-*co*-AAc)-5% AuNP (red circle) and P(AAm-*co0*-AAc)-10% AuNP (green triangle).

We have extracted the average elastic modulus plateau *G*’_o_ and the average critical deformation *γ*_0_, for each aqueous microgel dispersions at each studied concentration, and represented as a function of the concentration in Figure 6A,B. As it was expected, both *G*’_o_ and γ_0_, exhibit a power law relationship with the concentration that can be fitted to the form: *G*’_o_ ~ C^A^ and *γ*_0_ ~ C^B^. When evaluating the concentration dependence of *G*’_o_, positive slopes are observed for the studied microgel dispersions (as collected in Table 2). In contrast, the evolution of *γ*_0_ exhibit negative slopes.

According to the Shih et al. scaling model [31], the fact that *γ*_0_ exhibit a negative slope indicates that the three systems fall into the strong-link regime, in which inter-floc interactions are stronger than intra-floc. In the case of P(AAm-*co*-AAc)-5% AuNP microgel dispersion, this strong-link regime is also evidenced in the strain hardening depicted in Figure 5B for the dispersion containing 1 wt% of microgels. Thus, fractal dimension is obtained by applying Equations (1) and (2) of the Shih et al. model and collected in Table 2. But, as shown in the table, results obtained with the Shih et al. model are only valid for the P(AAm-*co*-AAc)-10% AuNP microgel dispersion, being this sample the one that fulfill the requirements mentioned above (having an *x* value lower than the fractal dimension and positive). In the case of P(AAm-*co*-AAc)-5% AuNP microgel dispersion, although 1 wt% dispersion clearly described a strain hardening behavior, thus supporting the strong-link regime, it is observed that upon addition of more microgels, 2 and 5 wt %, dispersions turn to strain thinning (see Figure 5B), which evidenced a transition between the two regimes, strong- and weak-link. Consequently, in order to evaluate the type of interactions Wu and Morbidelli’s scaling theory needed to be applied. From the applications of Equations (5)–(7) we could obtain the fractal dimension (Df) and microscopic elastic interaction regime (*α*) of the three studied microgel dispersions (Table 2). Some of the parameters showed significant experimental errors, in particular for P(AAm-*co*-AAc)-5% AuNP; these results would be more accurate with the study of more concentrations however, we have to indicate that our intention with this experiment was to obtain semiquantitative results for comparative purposes.

The estimation of *α* parameter resulted in values that evolves from 0.63 for P(AAm-*co*-AAc) to 0.04 for P(AAm-*co*-AAc)-10% AuNP, indicating an evolution from a transitioning regime toward a strong-link regime. It is worth mentioning that there is a lack of agreement between the semi-quantitative strong-link regime assessed for P(AAm-*co*-AAc)-10% AuNP microgel dispersion through Wu and Morbidelli’s model, and the qualitative shear thinning described in Figure 5C.

The encapsulation of AuNP within the microgel matrix contributes to increase the inter-floc interactions, or in other words, hampered the formation of cluster or agglomerates. This result is also in agreement with the estimation for the fractal dimension Df that varies from 2.14 to 1.2 with the incorporation of AuNP. This remarkable decrease of the fractal dimension also indicates that the incorporation of AuNP has changed the growth/aggregation mechanism of the aqueous dispersions. Fractal dimension is an indication of the growth mechanism and stacking density of the particles forming the floc. As stated in the literature two growing mechanisms are identified: reaction-limited and diffusion-limited aggregation mechanisms. Each mechanism gives rise to different agglomerates structures and thus, to different fractal dimensions. In the case of the reaction-limited mechanism, this ends up in agglomerates with denser structures (Df ~ 2.0–2.2) [47,48]. For the diffusion-limited mechanism, agglomerates form looser structures (Df ~ 1.7–1.8) [49]. Taking this into account, we can conclude that the incorporation of AuNP has modified the growth mechanism of P(AAm-*co*-AAc) micorgels, ending up in the formation of agglomerates with looser structures. It is worth mentioning that the obtained results could also be affected by the spatial distribution of the AuNP within microgel matrix that could be different as the content of AuNP increases [46]. This issue will be studied in further work.

## 4. Conclusions

The detailed rheological study performed in this work resolves that hybrid microgel dispersions present a gel-like behavior, besides confirming the elasticity reinforcement role of AuNP, as it was expected. By analyzing the behavior under deformation (strain sweep tests), we confirmed the colloidal gel structure which can break upon deformation but completely restructure as the deformation gradually disappears. Metal nanoparticles (AuNP) also play a major role in the recovery of the structure. From the application of the scaling theories, we also determined that the incorporation of metal nanoparticles (AuNP) affects significantly the colloidal structure formation to the point of modifying the growth mechanism from a reaction-limited to a diffusion-limited aggregation mechanism with the incorporation of 10 wt% AuNP. In conclusion, the incorporation of AuNP modifies the agglomerate growth mechanism giving rise to agglomerates with looser structures at rest that are easily deformable.

The strategy used in this work serves as a tool to control the formation, breakage, and reformation of agglomerates under stress through rheological characterization. Moreover, it opens a new way to predict metal nanoparticle effect in the microgel dispersions behavior through simple stress sweep tests, being the targeted objective. In addition, this facilitates the preparation of a more homogeneous and easy applicable system, for instance when applied for injectable systems [37]. In addition, the recovery of the elastic properties immediately after injection (deformation) may prevent the flow of the colloidal solution and facilitate that the material remains on the target site [50]. In summary, the approach presented herein is straightforward and simple, besides potentially inducing additional functionalities to the microgel system derived from the nanoparticles responsiveness itself.

Being able to control and program the flow behavior of microgel dispersion would be extremely helpful in order to ensure the success of their final application, in particular as drug delivery systems, carriers, or similar. Being able to tailor how stimuli responsive microgels should behave under deformation, and at rest, would avoid undesirable or unexpected agglomeration problems that could limit their responsiveness or have dramatic consequences as blood clots when referring to real in-vivo use of the smart dispersions. In conclusion, this work puts in evidence the importance of understanding the rheological behavior and fractal structure of stimuli responsive microgels for further potential applications that might be implied in flow/deformation of aqueous dispersions.

## Figures and Tables

**Table 1:** Recipe for the synthesis of the hybrid microgels

	AQUEOUS PHASE				ORGANIC PHASE	
	Acrylamide	Acrylic Acid	MBA	AuNP	Dodecane	Span 80
	(Mol)	(Mol)	(Mol)	(%wt)	(mL)	(g)
P(AAm*-co-*AAc)	0.056	2.77 × 10^−4^	1.29 × 10^−3^	0	30	0.5056
P(AAm*-co-*AAc)–5% AuNP	0.056	2.77 × 10^−4^	1.29 × 10^−3^	5	30	0.5056
P(AAm*-co-*AAc)–10% AuNP	0.056	2.77 × 10^−4^	1.29 × 10^−3^	10	30	0.5056

The volume of water in the aqueous phase is 10 mL. 1 wt% of AMPA-d intitiator was used.

**Table 2:** Summary of the results obtained by applying Shi et al., and Wu and Morbidelli’s fractal models.

Samples	Slopes (Figure 6A,B)		Shih et al.		Wu and Morbidelli			Regime
	A	B	*Df*	*x*	*Df*	*β*	*α*	
P(AAm*-co-*AAc)	2.56 ± 0.16	−0.22 ± 0.17	---	<0	2.14 ± 0.28	2.2 ± 0.5	0.63 ± 0.17	Transition (weak)
P(AAm*-co-*AAc)-5% AuNP	3.82 ± 0.78	−0.62 ± 0.22	---	<0	2.3 ± 0.7	2.3 ± 1.2	0.57 ± 0.38	Transition (weak)
P(AAm*-co-*AAc)-10% AuNP	2.29 ± 0.06	−1.18 ± 0.13	1.19	1.13	1.2 ± 0.4	4.14 ± 0.1	0.04 ± 0.03	Strong-link

## Supplementary Materials

The following are available online at https://www.mdpi.com/2079-4991/9/10/1499/s1, Figure S1: TGA Thermograms corresponding to P(AAm-*co*-AAc) (black), P(AAm-*co*-AAc)-5% AuNP (red) and P(AAm-*co*-AAc)-10% AuNP (green). Table S1: Results corresponding to the residue obtained from TGA experiments.
