# Supplementary material for: A Way to Predict Gold Nanoparticles/Polymer Hybrid Microgel Agglomeration Based on Rheological Studies
Source: Nanomaterials (Basel). 2019 Oct 21;9(10):1499. doi: 10.3390/nano9101499 (PMC6835908; doi:10.3390/nano9101499)
Supplement: Supplementary file 1 [file nanomaterials-09-01499-s001.pdf]

Supporting information for:

A Way to Predict Gold Nanoparticles/Polymer Hybrid Microgel Agglomeration Based on Rheological Studies

Coro Echeverría* and Carmen Mijangos

Institute of Polymer Science and Technology (ICTP-CSIC), C/Juan de la Cierva 3, 28006 Madrid, Spain; cmijangos@ictp.csic.es

* Correspondence: cecheverria@ictp.csic.es

Received: 10 September 2019; Accepted: 11 October 2019; Published: 21 October 2019

Thermogravimetric analysis.

In Figure S1 the thermogravimetric analysis corresponding to P(AAm-co-AAc) microgel and hybrid microgel containing theoretical 5 and 10 wt% AuNP is shown. As seen from the figure P(AAm-co-AAc) microgel sample described a three-step weight loss. (i) At temperatures below 200 °C, the weight loss is attributed to the loss of water adsorbed on the surface and/or trapped in the microgel. (ii) The second step appears at temperatures between 200 and 300 °C. This weight loss corresponds to the imidation reaction of the amide group [1]. (iii) Above 300–330 °C, the decomposition of the imide formed in the above degradation step takes place [1]. After the degradation process, the residue left is about 7%. When analyzing the hybrid microgels degradation process no significant differences are observed.

There are no appreciable differences between the reference sample and the hybrid microgel systems except for the residue left. As seen from the table, the samples P(AAm-co-AAc)-5% AuNP and P(AAm-co-AAc)-10% AuNP left 10 and 15% of residue, which implies a final AuNP content of 3 and 8%, respectively.

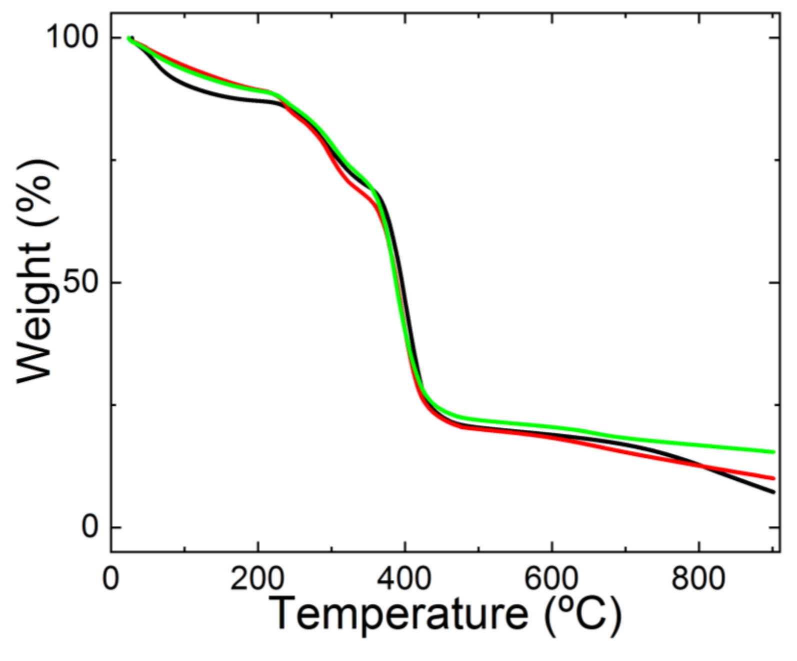

Figure S1. TGA Thermograms corresponding to P(AAm-co-AAc) (black), P(AAm-co-AAc)-5% AuNP (red) and P(AAm-co-AAc)-10% AuNP (green).

Table S1. Results corresponding to the residue obtained from TGA experiments.

SAMPLE	AuNP theoretical content (%)	TGA residue	AuNP experimental content (= TGA microgel residue–TGA Hybrid microgel residue)
P(AA-co-AAm)	0%	7%	
P(AAm-co-AAc)-5% AuNP	5%	10%	3%
P(AAm-co-AAc)-10% AuNP	10%	15%	8%

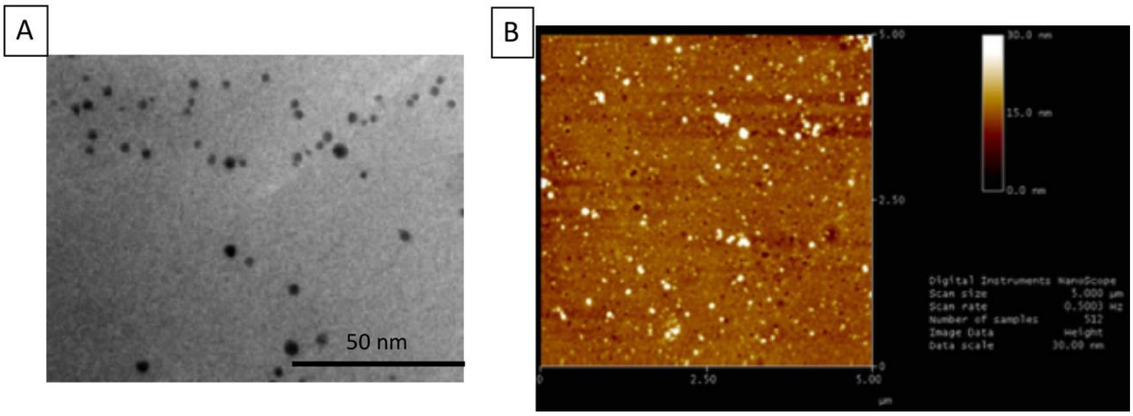

Figure S2. (A) TEM and (B)AFM images corresponding to as-received AuNP.

Reference:

1. Caulfield, M.J.; Qiao, G.G.; Solomon, D.H. Some aspects of the properties and degradation of polyacrylamides. *Chemical Reviews* **2002**, *102*, 3067–3084.
